# Hot spots and trends in PCI prognostic research: A bibliometric analysis with CiteSpace

**DOI:** 10.1097/MD.0000000000035599

**Published:** 2023-10-27

**Authors:** Shuli Guo, Xiandao Luo, Liu Huang, Changmin Wang, Yining Yang, Lei Yang

**Affiliations:** a Department of Health Management, School of Public Health, Hangzhou Normal University, Hangzhou, Zhejiang, China; b Clinical Laboratory Center, People’s Hospital of Xinjiang Uygur Autonomous Region, Urumqi, Xinjiang, China; c Department three of Cardiology, Urumqi Friendship Hospital, Urumqi, Xinjiang, China; d Department of Cardiology, People’s Hospital of Xinjiang Uygur Autonomous Region, Urumqi, Xinjiang, China.

**Keywords:** coronary artery disease, PCI, prognosis, research trends

## Abstract

**Background:**

The number of patients undergoing percutaneous coronary intervention (PCI) procedures is increasing along with the number of studies involving guidelines, prognostic assessments, and cardiac rehabilitation related to PCI strategies. However, fewer studies have reported the mapping of knowledge structure and hotspot analysis in this field. Our goal was to discuss and analyze the current status, hot spots and developmental trends associated with research into the prognosis of patients undergoing PCI, and to provide reference for PCI-related research.

**Methods:**

The Web of Science Core Collection and China Knowledge Network were searched for relevant literature from January 2003 to October 2022, and CiteSpace 6.1. R3 software was used to analyze the co-occurrence, clustering, and emerging authors, institutions, and keywords.

**Results:**

A total of 2666 English and 2010 Chinese publications were included. The number of publications showed a growing trend. The author with the maximum number of articles was Xu Bo. The institutions with high productivity were Peking Union Medical College and Capital Medical University. Although the number of Chinese articles was high, the cooperation between institutions was low and the impact was small. The results of the analysis suggest a shift in the focus of keywords from coronary artery disease and PCI to studies involving the assessment and intervention of risk factors associated with poor prognosis of PCI. Traditional Chinese Medicine and anxiety represent the emerging direction of PCI prognosis. The assessment of major adverse cardiovascular events and cardiac rehabilitation require careful analysis in post-PCI research.

**Conclusion:**

The findings of this bibliometric study present a comprehensive and systematic overview of the PCI prognosis, based on the analysis of the current status and trends in research, which may facilitate the identification of hot topics and new directions for future research.

## 1. Introduction

Cardiovascular disease is a major public health challenge worldwide, with China reporting the highest mortality rate due to the disease.^[1]^ Percutaneous coronary intervention (PCI) is the main treatment for coronary heart disease. A total of 1.04 million PCI procedures were performed in China in 2019, and the number of cardiac stents implanted were the highest in the world.^[2]^ Although PCI can alleviate symptoms caused by vascular stenosis, postoperative cardiac rehabilitation and outcomes are equally important. The PCI prognostic study refers to the studies investigating the prognosis or likely outcomes of patients who have undergone PCI, including those that affect the success of PCI and patient survival rates, and tracking patients to monitor their health outcomes. Despite extensive research studies investigating the prognostic value of PCI, fewer analyses of research hotspots have been reported in this field. Citespace software can be used to structure the detailed knowledge, visualize the complex relationships, and analyze the key trends based on the knowledge network structure with objectivity, systematicity, and scientific rigor.^[3]^ In this study, we utilized CiteSpace to conduct a bibliometric analysis of relevant literature to visualize the research hotspots in this field, in an effort to provide reference for subsequent prognostic studies involving PCI surgery.

## 2. Materials and Methods

### 2.1. Data sources and search strategies

**Figure 1.**
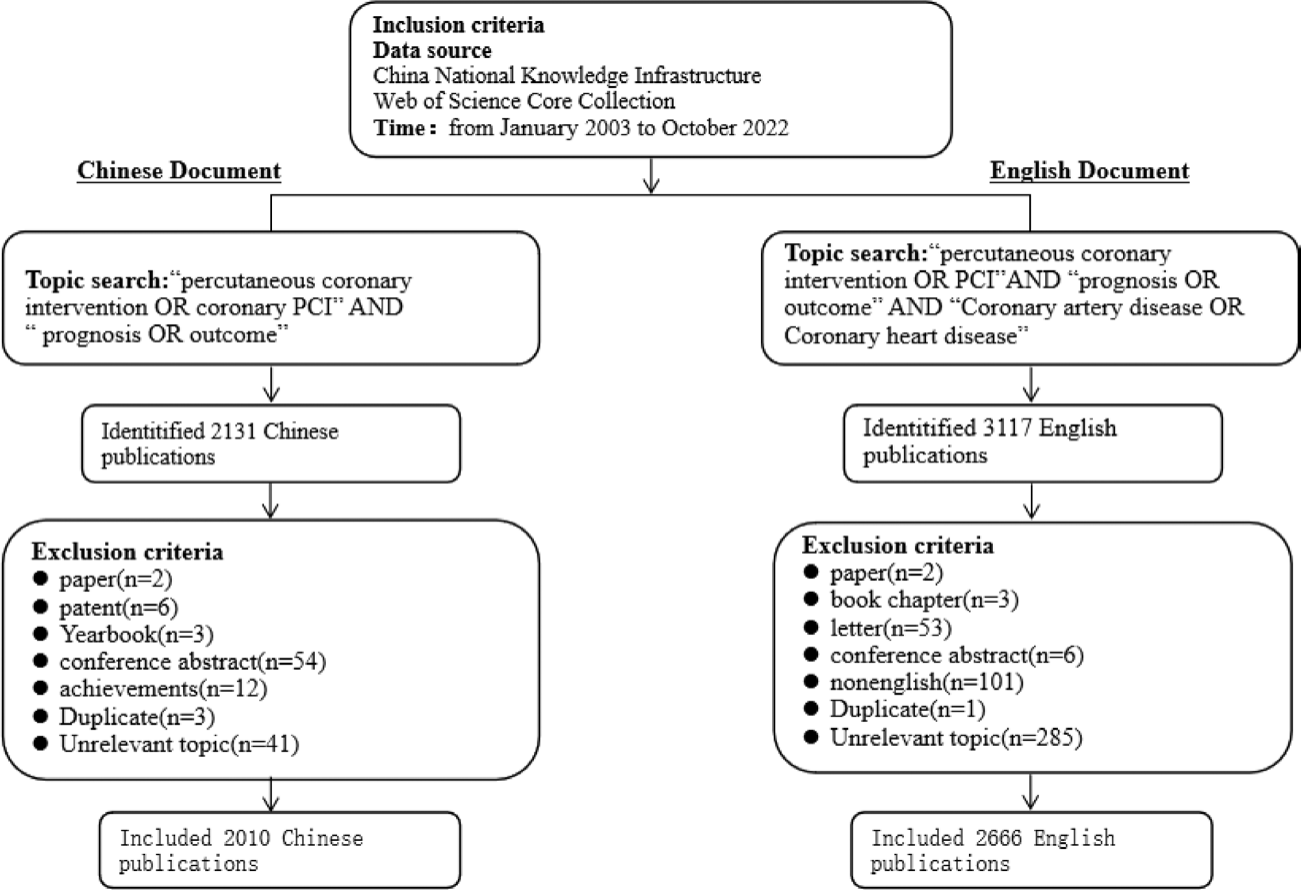
Flow chart of the literature selection.

Studies published between January 2003 and October 2022 were searched on November 10^th^,2022. The Chinese study data were obtained from the journal articles in the China National Knowledge Infrastructure using the search strategy of “Topic = percutaneous coronary intervention OR coronary PCI and Topic = prognosis OR outcome”. Published literature in English language were obtained from the Science Citation Index Expanded of the Web of Science Core Collection (WoSCC) using the search strategy of “Topic = (percutaneous coronary intervention OR PCI) AND Topic = (prognosis OR outcome) AND Topic = (Coronary artery disease OR Coronary heart disease)”. A total of 2131 Chinese and 3117 English publications were retrieved, which were exported in Refworks format in Chinese and in full record plain text format in English. The study records included title, author, institution, keywords, year, and journal. Substandard documents were excluded and then analyzed. To ensure the accuracy of the study data, the search process was conducted independently by 2 researchers by searching, checking, screening, and confirmation. Details of the filtering process are presented in Figure 1.

### 2.2. Data analysis

SPSS 26.0 (SPSS Inc, Chicago, IL) was used to create the fitting curve for the number of publications. CiteSpace (version 6.1.R3, Drexel University, Philadelphia, PA, https://citespace.podia.com/download) was used to analyze the publications that contributed to the prognosis of PCI. First, the source terms included author, title, keywords, and abstract, while the node type was set as institution, country, cited journal or keyword. Second, the cosine link reduction method and pathfinder networking scaling were utilized to simplify the network and highlight the important features. Third, we performed a keyword co-occurrence and cluster analysis by combining similar objects to determine related areas of study. We used the log-likelihood ratio algorithm to analyze related terms and keywords associated with PCI prognosis. The number of objects involved in the cluster were determined according to the size, and the largest clusterID 0 (#0) contained the largest number of keywords. The silhouette value (S) reflected the similarity of a term to members in the cluster, with S > 0.7 indicating a high degree of consistency. Fourth, we used the burst detection function to explore the emerging trends during a specific time period. For visualization, rings of different colors in the node represent the node frequency of occurrence in different years. Centrality is an indicator that can be used to evaluate the importance of the item in the network. Generally, the purple outer circle shows the term with centrality ≥ 0.1, indicating the strong interactive significance of the node.

## 3. Results

### 3.1. Distribution of annual publications

**Figure 2.**
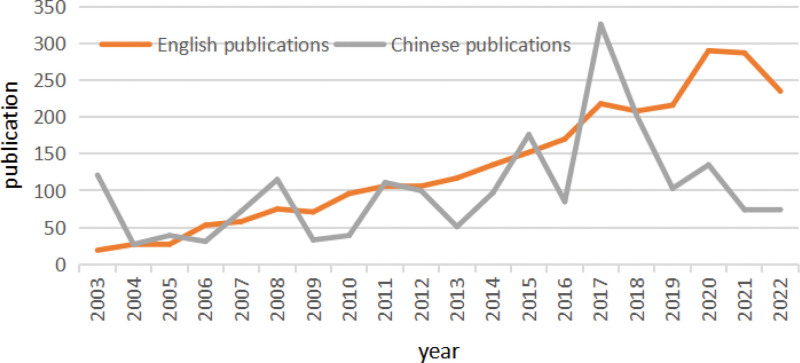
Annual number of Publications related to PCI prognosis. PCI = percutaneous coronary intervention.

A total of 2131 Chinese and 3117 English publications from 2003.1.1 to 2022.10.31 were extracted from China National Knowledge Infrastructure and WoSCC. The search process is shown in Figure 1. The final analysis included 2010 Chinese studies and 2666 English records. Since 2003, the number of publications in Chinese associated with PCI prognosis fluctuated around 100 annually, with a maximum of 326 articles in 2017. The publications in English increased nearly every year since 2003, with a peak of 290 articles published in 2020 (Fig. 2).

### 3.2. Bibliometric analysis of authors, institutions, and countries

**Figure 3.**
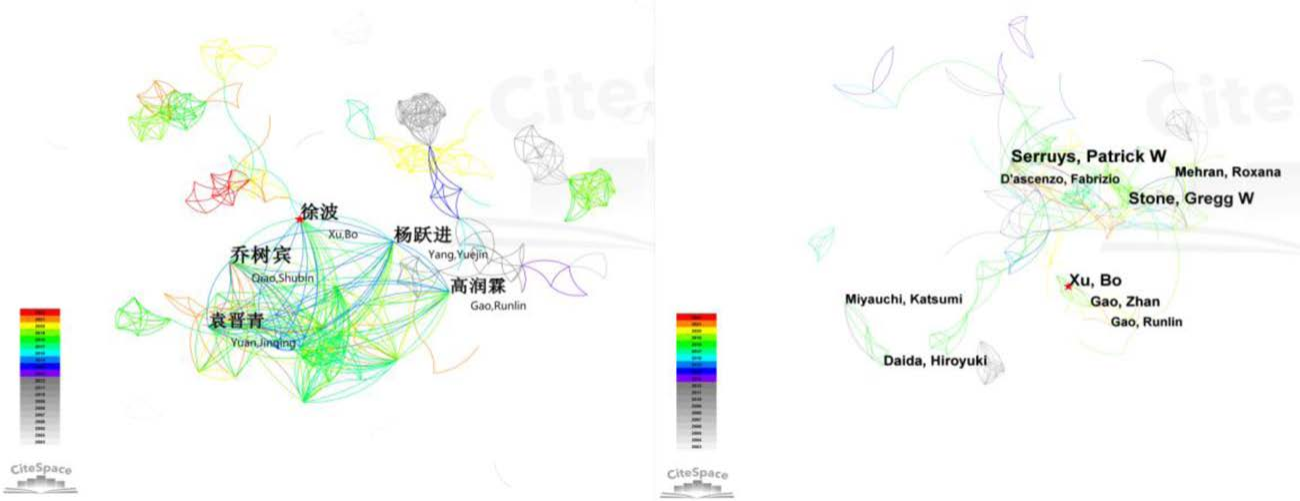
Bibliometric analysis of authors related to PCI prognosis research (A) Chinese publications, (B) English publications. PCI = percutaneous coronary intervention.

The Chinese publications involved 730 authors. Qiao Shubin was the most cited (50 times) Chinese author, followed by Xu Bo. the publications in English involved 864 authors, and the top 2 cited authors were Serruys, Patrick W (37 times) and Xu Bo. The number of authors with 1 publication in Chinese was 270 (37.0%), which was higher than those in English (141 or 16.32%). The author with the most citations in English and Chinese combined was Xu, Bo from Fu Wai Hospital, Chinese Academy of Medical Sciences. According to the author cooperation network relationship map (Fig. 3), Chinese authors formed 736 nodes (N) and 1419 lines (E). Authors in English had relatively close connection (N = 864, E = 2138). Table 1 shows the Top 5 active authors.

**Table 1:** Top 5 active authors in the research.

Chinese publications					English publications				
Rank	Publications	Been Cited	Year	Author	Rank	Publications	Been Cited	Year	Author
1	20	37	2003	乔树宾Qiao Shubin	1	34	50	2007	Serruys Patrick W
2	14	32	2015	徐波 Xu Bo	2	25	42	2014	Xu Bo
3	14	30	2014	杨跃进 Yang Yuejin	3	24	39	2014	Stone Gregg W
4	13	18	2015	高润霖 Gao Runlin	4	18	35	2018	Gao Zhan
5	8	18	2011	袁晋青Yuan Jinqing	5	15	34	2008	Daida Hiroyuki

**Figure 4.**
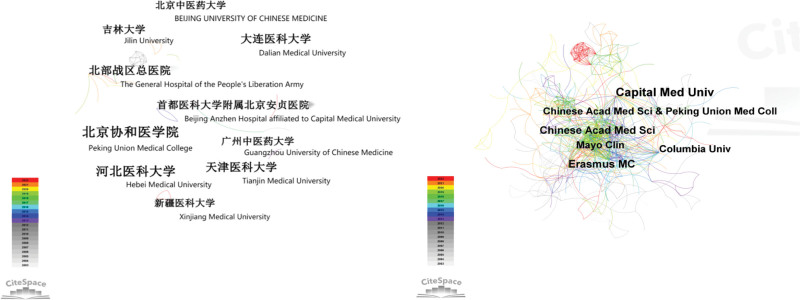
Knowledge map of institutions related to PCI prognosis research. (A) Chinese publications, (B) English publications. PCI = percutaneous coronary intervention.

**Figure 5.**
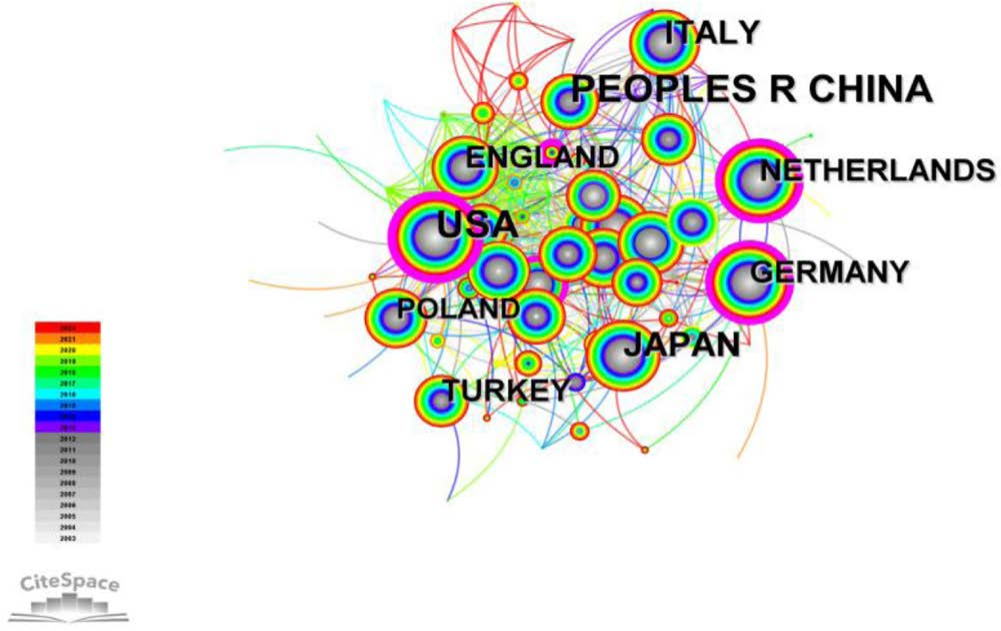
Knowledge map of countries related to PCI prognosis research. PCI = percutaneous coronary intervention.

As showed in Figure 4, the publications in Chinese involved a total of 478 institutions (N = 478, E = 150), and the top 5 institutions in terms of number of publications are shown in Table 2. Articles published in English involved 609 institutions with a stronger network of cooperation (N = 609, E = 1223). The articles in English covered 91 countries. In terms of frequency, the top 3 countries were China, the United States, and Japan (Fig. 5, Table 3). Since 2003, the number of publications in China increased in a nearly linear fashion, with a maximum of 121 publications in 2021.As shown in Figure 5,the larger the node, the more publications is available under that country, The connections or links between nodes/terms represents the number of times terms occur together in the same country, and the width of the connection indicates the degree of cooperation. However, unlike the USA and the Netherlands, China does not have a purple outer ring. The purple outer circle indicates that the node has a high intermediary centrality, >0.1, which plays a bridging role with a relatively significant influence in this area.

**Table 2:** Top 5 institutions contributed to the publications.

Chinese publications				English publications			
Rank	Been cited	Year	Institution	Rank	Been cited	Year	Institution
1	49	2008	北京协和医学院Peking Union Medical College	1	92	2008	Capital Med Univ
2	41	2004	河北医科大学Hebei Medical University	2	61	2003	Erasmus MC
3	26	2006	天津医科大学Tianjin Medical University	3	55	2013	Chinese Acad Med Sci
4	25	2019	北部战区总医院the General Hospital of People’s Liberator Army	4	43	2008	Mayo Clin
5	24	2007	大连医科大学Dalian Medical University	5	42	2005	Columbia Univ

**Table 3:** Top 10 countries contributed to the publications.

Rank	Been cited	Centrality	Year	Country
1	599	0.01	2003	Peoples R China
2	539	0.32	2003	USA
3	294	0.01	2003	Japan
4	194	0.06	2003	Italy
5	153	0.11	2003	Netherlands

### 3.3. Bibliometric analysis of co-cited references and top-cited articles

**Figure 6.**
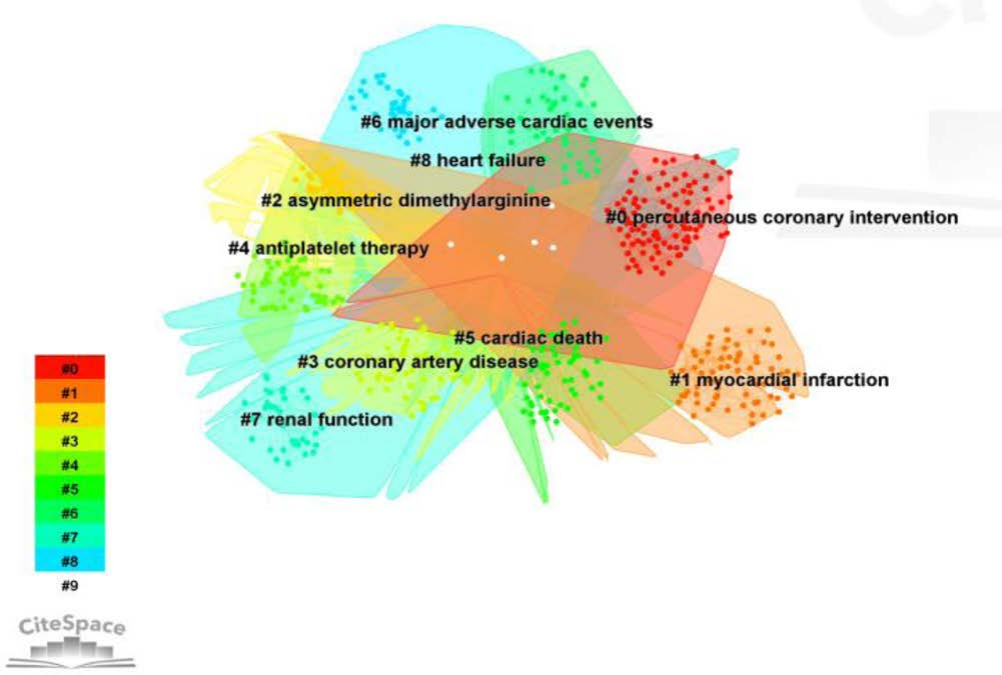
Knowledge map of clustering of co-cited references on PCI prognosis. PCI = percutaneous coronary intervention.

Analysis of the higher co-cited references provides access to the disciplinary research base (Table 4). The 2017 ESC guidelines for the management of patients with acute myocardial infarction with ST-segment elevation were the most frequently cited (119 times in total). The ESC/EACTS guidelines for myocardial remodeling and optimal pharmacotherapy with or without PCI in stable coronary artery disease had a higher mediated centrality of 0.14 and 0.13, respectively, and these 3 articles represent important references for the development of PCI treatment. Some of the literature is closely related and is often cited together with the cited literature, that is, these documents often appear together in multiple documents published later. It also means that these co-cited documents have some similarities in content. From this, further clustering is carried out to explore the common theme of similar literature. Cluster analysis of co-cited literature was conducted and sorted according to the number of members and citations under different classifications. The smaller the cluster ID, the higher the number of members and the greater the influence. Figure 6 revealed 7 main directions of prognostic research focus on coronary PCI at this stage, which lays the foundation for prognostic research on PCI.

**Table 4:** Top 10 co-cited references on PCI prognosis.

Rank	Frequency	Centrality	Year	Cited references
1	119	0.03	2018	2017 ESC Guidelines for the management of acute myocardial infarction in patients presenting with ST-segment elevation.
2	74	0.14	2014	2014 ESC/EACTS Guidelines on myocardial revascularization: The task force on myocardial revascularization of the European Society of Cardiology (ESC) and the European Association for Cardio-Thoracic Surgery (EACTS) developed with the special contribution of the European Association of Percutaneous Cardiovascular Interventions (EAPCI)
3	72	0.09	2016	2015 ESC guidelines for the management of acute coronary syndromes in patients presenting without persistent ST-segment elevation: Task force for the management of acute coronary syndromes in patients presenting without persistent ST-Segment Elevation of the European Society of Cardiology (ESC).
4	61	0.01	2019	2018 ESC/EACTS guidelines on myocardial revascularization.
5	58	0.03	2012	ESC guidelines for the management of acute myocardial infarction in patients presenting with ST-segment elevation.
6	49	0.01	2020	2019 ESC guidelines for the diagnosis and management of chronic coronary syndromes.
7	38	0.05	2019	Fourth universal definition of myocardial infarction (2018).
8	36	0.04	2009	Percutaneous coronary intervention versus coronary artery bypass grafting for severe coronary artery disease.
9	31	0.13	2007	Optimal medical therapy with or without PCI for stable coronary disease.
10	31	0.01	2020	Initial invasive or conservative strategy for stable coronary disease.

PCI = percutaneous coronary intervention.

### 3.4. Bibliometric analysis of co-occurring keywords

#### 3.4.1. Co-occurrences, and cluster analysis of keywords.

**Figure 7.**
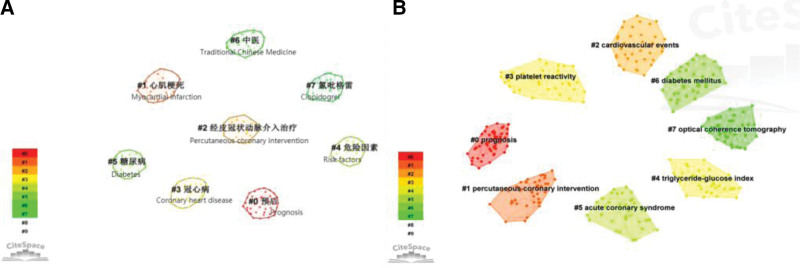
Knowledge map of clustering of keywords on PCI prognosis. (A) Chinese publications, (B) English publications. PCI = percutaneous coronary intervention.

**Figure 8.**
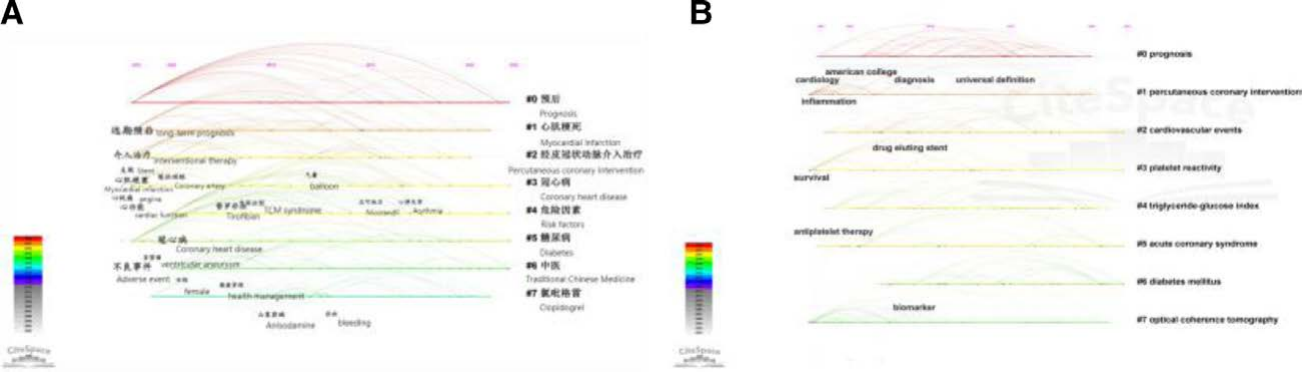
Timeline of clustering of keywords on PCI prognosis. (A) Chinese publications, (B) English publications. PCI = percutaneous coronary intervention.

A keyword is a high overview and summary of an article; the frequency of keywords usually indicates the focus of the field. The knowledge map of Chinese publications contains 476 keywords and English publications contain 637 keywords, Table 5 shows the general consistency of top 20 keywords in Chinese and English. The knowledge map of keyword clusters of PCI prognosis is presented in Figure 7 and Table 6. The Chinese and English publication clusters of keywords were reasonable (Chinese: Modularity = 0.8748, S = 0.979; English: Modularity = 0.742, S = 0.8932), indicating a high degree of consistency among members within each category of clusters, with valid and convincing results. Both English and Chinese publications focused primarily on prognostic studies based on cardiovascular events, diabetes, and antiplatelet therapy. While Traditional Chinese Medicine (TCM) was the focus in Chinese publications, the English publications dwelt primarily on triglyceride-glucose index and optical coherence tomography (OCT)-guided PCI-related research. The keyword timeline plots (Fig. 8) reveal hot keywords in the Chinese and English literature ranging from coronary artery disease and PCI transfer to studies on prognosis, cardiovascular events, risk factors, and interventions.

**Table 5:** Top 20 keywords in Chinese and English articles on PCI prognosis.

Rank	Chinese publications			English publications		
	Frequency	Centrality	Keyword	Frequency	Centrality	Keyword
1	681	0.55	预后prognosis	1151	0.02	Percutaneous coronary intervention
2	315	0.34	不良事件Adverse events	787	0.02	Coronary artery disease
3	264	0.31	冠心病Coronary heart disease	555	0.01	Myocardial infarction
4	105	0.21	冠脉支架Coronary stents	542	0	Mortality
5	76	0.16	危险因素Risk factors	460	0.01	Risk factor
6	56	0.1	替罗非班Tirofiban	421	0.01	Glomerular filtration rate
7	55	0.03	心功能Cardiac function	408	0.01	Acute myocardial infarction
8	50	0.03	糖尿病Diabetes	398	0.02	Prognosis
9	48	0.08	氯吡格雷Clopidogrel	387	0.02	Cardiac Rehabilitation
10	41	0.04	预后评估Prognostic evaluation	330	0.01	Major adverse cardiovascular events
11	40	0.04	冠状动脉Coronary artery	313	0.02	Cardiovascular disease
12	39	0.13	介入治疗Interventional therapy	287	0.01	Society
13	37	0.05	健康管理Health management	270	0.03	Risk intervention
14	32	0.02	焦虑 Anxiety	269	0.03	Acute coronary syndrome
15	26	0.02	球囊Balloon	254	0.03	Heart failure
16	23	0.01	血栓抽吸Thrombus aspiration	252	0.02	Triglyceride-glucose index
17	23	0.02	老年人the elderly	218	0.02	Health management
18	21	0	性别Gender	199	0.03	Revascularization
19	21	0.02	心脏康复Cardiac Rehabilitation	178	0.02	Angioplasty
20	19	0.05	中医Traditional Chinese Medicine	171	0.01	Vascular endothelial function

PCI = percutaneous coronary intervention.

**Table 6:** Clustering labels of keywords of Chinese and English publications on PCI prognosis.

ClusterID	Chinese publications			English publications		
	Size	Silhouette	Label (log-likelihood ratio, p level)	Size	Silhouette	Label (log-likelihood ratio, p level)
0	57	0.864	预后(124.61, 1.0E–4) Prognosis	77	0.762	Prognosis (2867.3, 1.0E–4)
1	57	0.798	心肌梗死 (66.57, 1.0E–4) Myocardial infarction	74	0.819	Percutaneous coronary intervention (1555.72, 1.0E–4)
2	48	0.842	经皮冠状动脉介入治疗 (57.75, 1.0E–4) Percutaneous coronary intervention	61	0.791	Cardiovascular events (1899.78, 1.0E–4)
3	47	0.803	冠心病 (127.77, 1.0E–4) Coronary heart disease	58	0.793	Platelet reactivity (3753.15, 1.0E-4)
4	41	0.881	危险因素 (51.85, 1.0E–4) Risk factors	58	0.77	Triglyceride-glucose index (7666.16, 1.0E–4)
5	31	0.892	糖尿病 (51.68, 1.0E–4) Diabetes	55	0.776	Acute coronary syndrome (2967.22, 1.0E–4)
6	25	0.926	中医(36.49, 1.0E–4) Traditional Chinese Medicine	46	0.741	Diabetes mellitus (5243.12, 1.0E–4)
7	25	0.927	氯吡格雷 (83.89, 1.0E–4) Clopidogrel	43	0.726	Optical coherence tomography (2672.71, 1.0E–4)

PCI = percutaneous coronary intervention.

#### 3.4.2. Burst detection of keywords.

**Figure 9.**
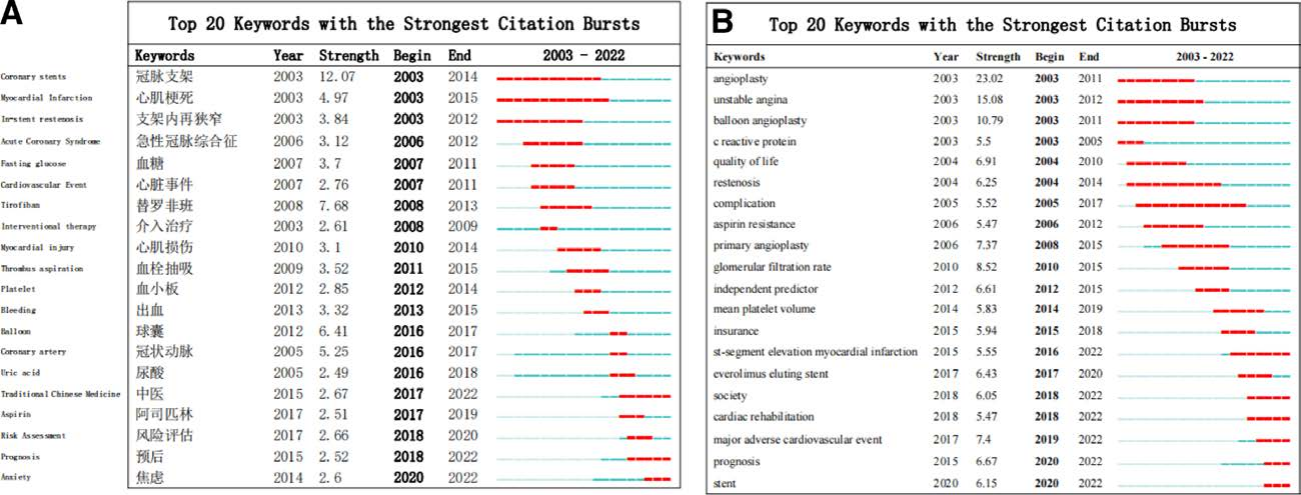
TOP 20 keywords with the strongest citation bursts on PCI prognosis. (A) Chinese publications, (B) English publications. PCI = percutaneous coronary intervention.

Figure 9 presents the 20 keywords and hotspots with citation bursts over the last 20 years. Among the Chinese publications, “myocardial infarction” appeared the earliest and lasted the longest (2003–2015). The strongest burst term was “coronary stent,” while TCM, prognosis, and post-PCI anxiety were emphasized from 2017 to 2020, consistently until 2022, which are the current research hotspots. The longest duration of keywords in English publications was complication (2005–2017) and the strongest burst term was angioplasty. ST-segment elevation myocardial infarction, society, cardiac rehabilitation, major adverse cardiovascular events, prognosis, and stents emerged in the last 6 years and have been consistently reported until 2022, and are the current research hotspots.

## 4. Discussion

More than 11.39 million patients are currently diagnosed with coronary heart disease in China, and the country reports the highest mortality rate worldwide due to this disease.^[4]^ PCI can be used to rapidly open up occluded coronary arteries and restore blood flow and myocardial reperfusion. It is one of the most effective clinical treatments for coronary artery disease. However, PCI does not change the disease pathophysiology. Major adverse cardiovascular events (MACE such as restenosis and death) can still occur after PCI, with an incidence reaching as high as 30.5% at 5 years after PCI.^[5]^ Prognostic assessment and intervention of patients undergoing PCI is important to consolidate the efficacy of surgery and improve patient survival and quality of life. With the increase in the incidence of coronary artery disease, advances in treatment technology, guidelines continue to be introduced and treatment is constantly standardized, improvements in medical coverage, and reduction in the price of stents, more and more people are willing to accept PCI, the number of PCI procedures has increased with a growing emphasis on PCI prognosis.^[6]^ In addition, the aging population in China is an increasingly serious concern, the number of elderly patients with coronary heart disease continues to increase. The relevant knowledge mapping inter-author, inter-institutional, and inter-country linkage (Figs. 3–5) showed that PCI prognosis research has formed a certain cooperative network in China, the citation frequency of the relevant literature (Table 3) also indicates to some extent that Chinese literature has provided a reference for related research in this field and promoted the development of the discipline of cardiac rehabilitation post-PCI.

China has focused increasingly on PCI, with a comprehensive investigation in related fields, independently developing bioresorbable scaffolds, and practicing the concept of “intervention without implantation” for vascular reconstruction.^[7]^ Meanwhile, the National Center for Cardiovascular Quality Improvement and the National Quality Control Center for Coronary Heart Disease have strengthened the quality of cardiovascular disease management and improved the quality and service levels. China’s efforts in the treatment of coronary heart disease using PCI serve as a standard of reference for other countries in improving human health.

In this study, a summary analysis of related articles in the last 20 years revealed a steady upward in the overall number of publications associated with PCI prognosis since 2003.The main reasons include not only extensive PCI procedures performed in China, the PCI-related guidelines have also contributed to the continuous standardization of PCI diagnosis and treatment. For example, the guidelines and expert consensus on ST-segment elevation myocardial infarction/non-ST elevation myocardial infarction, countermeasures for post-PCI bleeding, and management options for complete obstruction have been issued.^[8–10]^ In 2002, China also successively issued PCI-related treatment guidelines, and later released different versions in 2009, 2012, and 2016.^[11–14]^ The peak of Chinese literature in PCI occurred in 2017 immediately afterwards (Fig. 2). These guidelines further promoted the increase in PCI procedures and related research.

A strong cooperative network has been formed among the authors, and a core group of authors associated with PCI prognostic studies has been established. There is less cooperation among Chinese institutions or authors, with the proportion of authors publishing a single article as high as 37%. Most of the research institutions are hospitals or universities, without leading participation at the national or governmental level. They represent single research entity, with no obvious clustering among institutions. China is the country with the highest frequency of publications in this field, indicating increased attention and active exploration in PCI prognosis. However, compared with the United States and The Netherlands, China is less central and less influential. It is necessary to further strengthen the cooperation between institutions and countries to further deepen the research direction, to play a bridging role in studies investigating PCI prognosis.

By clustering the co-cited literature and an in-depth analysis of important literature, we found that the current focus of prognostic studies involving PCI in coronary artery disease is 3-fold: Consensus or guidelines related to ST-segment elevation myocardial infarction and coronary revascularization. The guidelines based on clinical effectiveness trials have clarified the criteria for the selection of revascularization strategies, the intraoperative operation of PCI and the prevention and treatment of major complications of PCI, the antithrombotic treatment in the perioperative period of PCI and the postoperative management;^[15–18]^ The population benefiting from PCI is still not clearly identified. Further studies are needed to confirm the incidence of poor cardiovascular events after PCI compared with those before PCI. PCI and pharmacotherapy are the best treatment options for stable coronary artery disease, and the classification of patients with myocardial infarction needs to be further optimized;^[19–21]^ Studies related to the prognosis of MACE after PCI. The main focus is on the risk factors associated with the occurrence of MACE (including cardiac-caused death, all-cause death, in-stent thrombosis, restenosis, and revascularization) after PCI, and the effectiveness of different interventions to improve the prognosis.^[22,23]^

Based on cluster analysis of keywords, we found that TCM emerged in Chinese publications, while English publications focused on triglyceride-glucose index and OCT-guided PCI-related research. PCI treatment for coronary heart disease was adopted early in foreign countries, and surgical techniques are constantly improving. OCT for the management of complex bifurcation lesions has advantages in guiding accurate PCI treatment.^[24]^ Cardiac rehabilitation has been carried out early in foreign countries with medical insurance support, and sufficient attention has been paid to the study of PCI prognosis. The “metabolic comorbidity problem” can significantly affect the prognosis of PCI and is also a treatment difficulty, making it a research hotspot.^[25]^ TCM provides a systematic diagnosis and treatment of coronary heart disease. In recent years, evidence-based medicine has been continuously developed, and research has shown that TCM and other factors can affect the prognosis of PCI. Further, Baduanjin and others have reported strong results in cardiac rehabilitation after PCI, laying the foundation for the study of TCM in the prognosis of PCI.^[26,27]^

Based on keyword frequency, centrality, clustering labels, keyword emergence time and intensity and comparison with foreign countries, we analyzed the 3 main directions of PCI prognosis research in China: Immediate, intermediate and long-term prognostic assessment of patients with PCI. Risk assessment models and risk scores are often used for prognostic assessment of PCI.^[28–30]^ The main risk factors include age, gender, history of diabetes, treatment modality (thrombolysis, OCT guidance),^[31,32]^ platelet activity,^[33]^ triglyceride-glucose index,^[34]^ lesion vascular condition and other indicators^[35]^; Cardiac rehabilitation (CR) and health management of patients after PCI. CR significantly reduces cardiovascular death and all-cause mortality, improves exercise tolerance, and benefits almost all patients with coronary artery disease. It is an important adjunct to PCI treatment. The CR referral rate for patients after PCI has reached about 60% in USA, with dedicated CR facilities and relatively mature procedures that are covered by health insurance.^[36]^ CR is based on exercise in a variety of forms, and is traditionally used mostly in medical institutions and family-centered exercise interventions. Community-based and information technologies such as AI-guided CR have proven effective.^[37–40]^ Behavioral management of patients undergoing PCI using diet and exercise has significantly improved patients quality of life;^[41,42]^ Management of risk factors after PCI. Anxiety and depression occur in approximately 21.5% of patients 24 hours after PCI,^[43]^ which can affect the long-term survival of patients and the incidence of MACE after PCI.^[44–46]^ Studies have used psychological counseling and other methods to alleviate patients anxiety and depression and improve disease prognosis.^[47,48]^ Treatment using TCM-based interventions emerged in 2017 to 2019 alone or in conjunction with Western medicine, to facilitate “double heart recovery,” relieve anxiety and reduce the occurrence of MACE.^[49–52]^ In addition, TCM exercises such as Tai Chi Chuan and Ba Duan Jin have also been shown to improve PCI prognosis. Further evidence is expected to support the role of TCM in improving the prognosis of PCI.^[27,53,54]^

In conclusion, compared with foreign countries, China has made multi-faceted exploration in the field of PCI prognosis, with concrete achievements. The future further depends on several factors. First, based on the current scenario, research at each institution is relatively independent and scattered, but the cooperation and communication between research subjects has increased. Second, to focus on hot spots, the research content needs to be deepened, and a sound and standard prognostic assessment system for PCI needs to be established. Third, interdisciplinary cross-fertilization is required to build a personalized health management program for patients post-PCI. Fourth, the randomized controlled studies of PCI prognostic interventions should be conducted to lay the foundation for the development of guidelines related to PCI prognosis.

## 5. Conclusion

We conducted a systematic review and bibliometric analysis of studies related to PCI prognosis from 2003 to 2022, which provided important insights into research trends, hotspots, and future directions. An overall upward exists in the number of publications reporting PCI prognosis. China has published a limited number of articles in this research area. However, there is a need to strengthen cooperation, deepen the research content, and improve the impact. In addition, the research frontiers and trends in PCI prognosis may focus on MACE assessment, CR, and risk factor intervention. This bibliometric analysis summarizes and distinguishes the research hotspots and frontiers as well as the corresponding directions, provides effective strategies and enlightenment for future studies on PCI prognosis.

## 6. Strengths and limitations

The analysis of Chinese and English publications involving PCI prognosis in the past 20 years has practical implications in helping investigators in China to comprehend the research hotspots and the latest progress in this field, establish the direction of scientific research, and improve the quality and impact of research.

Only a limited number of articles and reviews published in English were included in the WoSCC database, which may lead to inaccurate and biased results. However, the overall scientific merit of the study results and conclusions is not affected.

## Acknowledgments

We would like to thank ChaoMei Chen from Drexel University, USA, for developing the CiteSpace software and making it available online for free.

## Author contributions

**Conceptualization:** Changmin Wang, Lei yang.

**Data curation:** Liu Huang.

**Formal analysis:** Yining Yang.

**Writing – original draft:** Shuli Guo.

**Writing – review & editing:** Xiandao Luo.
